# Neuroimmunomodulation of AMPA Receptors Through Combination of Medium-Chain Triglycerides and Perampanel to Treat Status Epilepticus in Anti-NMDA Receptor Encephalitis

**DOI:** 10.1007/s11481-026-10295-w

**Published:** 2026-05-19

**Authors:** Laura Marie Stadler, Laurin Schappe, Piergiorgio Lochner, Luna Bonifer, Mathias Fousse, Sven G. Meuth, Sergiu Groppa, Yaroslav Winter

**Affiliations:** 1 Department of Neurology, Saarland University Medical Center, University of Saarland, Kirrberger Str. 100, Homburg, D-66421 Germany; 2 Department of Neurology, University Hospital Münster, Münster, Germany; 3 Department of Neurology, Philipps-University Marburg, Marburg, Germany

**Keywords:** Medium-chain triglycerides, Perampanel, Status epilepticus, Anti-NMDA receptor encephalitis, Autoimmune encephalitis

## Abstract

Recent studies have shown that a ketogenic diet containing medium-chain triglycerides (MCTs) has a synergistic effect with perampanel in modulating AMPA receptors. Both MCTs and perampanel have immunomodulatory properties. However, the use of the combination of MCTs and perampanel to treat status epilepticus caused by autoimmune encephalitis has never been investigated. After multiple antiseizure (benzodiazepines, levetiracetam, brivaracetam, lacosamide, ketamine, propofol, and isoflurane) and immunomodulatory treatments (glucocorticoid pulse (days 4–8), intravenous immunoglobulin (days 10–12), plasmapheresis (days 13–19), rituximab (days 22 and 34), cyclophosphamide (day 38)), failed to interrupt status epilepticus in a 33-year-old woman caused by paraneoplastic anti-N-methyl-D-aspartate (NMDA) receptor encephalitis, we initiated perampanel treatment (12 mg/day), which significantly reduced ictal activity in the EEG. Status epilepticus was finally terminated when MCTs were added to the perampanel treatment regimen. Our clinical data may support previous in vitro studies demonstrating that combining perampanel with MCTs improves the neuroimmunomodulatory effect on AMPA receptors. MCTs may have a synergistic effect when added to perampanel to treat autoimmune status epilepticus. The possible cumulative effects of combination with other immune therapies in our case cannot be completely excluded.

**Graphical Abstract:**

**Figure:**
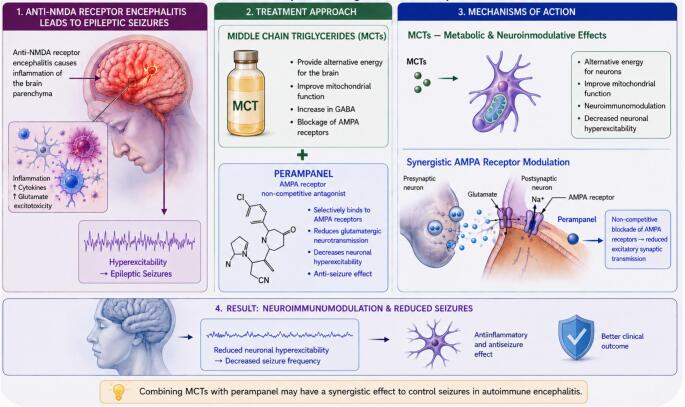
Source: OpenAI (ChatGPT) 15 May 2026

## Introduction

Super-refractory status epilepticus is defined as status epilepticus that continues for at least 24 h after the start of anaesthetic therapy, or that recurs after anaesthesia is stopped. Autoimmune encephalitis is a frequent etiology of super-refractory status epilepticus and requires an adequate combination of antiseizure medication and immune modulation considering synergistic effects of applied treatments (Vasquez et al. 2019).

The ketogenic diet (KD) is a high-fat, low-carbohydrate diet whose effectiveness in treating drug-resistant epilepsy is well-known (Shin et al. 2025). KD also has the potential to modulate the immune system, specifically the metabolism of immune cells (Guerreiro et al. 2024). The C10-enriched medium-chain triglyceride (MCT) KD is a modified form of the KD. Unlike the classic KD, which is high in fat and low in carbohydrates and protein, the MCT KD contains higher proportions of carbohydrates and protein (Dutta et al. 2022). It has also been found that MCT modulates immune responses (Yu et al. 2017, 2019). Published data indicates that MCTs suppress macrophage phagocytosis and downregulate the production of IL-6 and TNF-α, the transcription of COX-2 and iNOS, and the expression of CD80 on cell surfaces (Yu et al. 2017, 2019). Compared with a common long-chain triglyceride diet, MCT shows potential for a greater antiseizure effect (Prasoppokakorn et al. 2019). The reasons for this are twofold: indirect effects such as improved mitochondrial function and modulation of astrocyte activity, and direct effects through non-competitive inhibition of α-amino-3-hydroxy-5-methyl-4-isoxazolepropionic acid receptor (AMPA) receptor activity (Shin et al. 2025). AMPA receptor modulation is used in the treatment of epilepsy, for example with perampanel, which is a selective, non-competitive AMPA receptor antagonist. This reduces the excitability of neurons caused by glutamate (Hibi et al. 2012). A recent study has also shown that perampanel has immunomodulatory properties (Wasser et al. 2025). Due to the various modulation points of the AMPA receptor, it is assumed that KD and perampanel have a synergistic effect (Falsaperla et al. 2025). The following case report describes a young woman with super-refractory status epilepticus due to anti-N-methyl-D-aspartate (anti-NMDA) receptor encephalitis, which resolved following treatment with a combination of medium-chain triglycerides and the AMPA receptor antagonist perampanel.

## Methods

### Clinical Evaluation

**Fig. 1:**
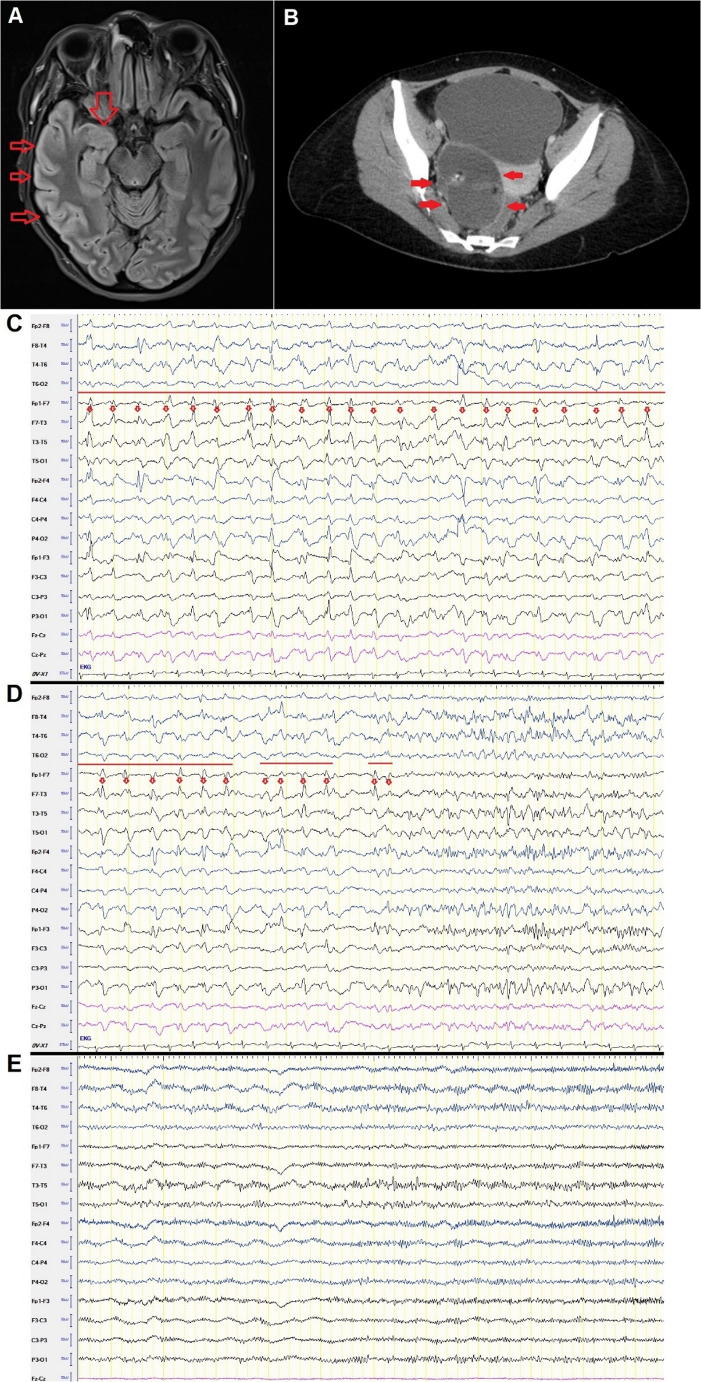
**A**: The arrows indicate a T2 hyperintensity in the right temporal lobe, which is consistent with anti-NMDA receptor encephalitis. **B**: The arrows indicate a right ovarian teratoma. **C**: EEG recording shows secondary generalized status epilepticus with the origin in the fronto-central area. A red line depicts the duration of ictal activity, while red dots indicate epileptiform discharges; **D**: EEG shows intermittant interruptions of ictal activity since the initiation of perampanel treatment. A red line depicts the duration of ictal activity, while red dots indicate epileptiform discharges; **E**: status epilepticus was terminated after medium-chain triglycerides were added to perampanel

A 33-year-old woman was transferred to the intensive care unit of the Department of Neurology at Saarland University Medical Centre from a peripheral hospital due to refractory status epilepticus. Nine days earlier, she had been admitted to the primary hospital due to headaches and behavioural abnormalities. The cranial MRI scan showed a T2 hyperintensity in the right temporal lobe (Fig. 1A). The lumbar puncture revealed pleocytosis of 38 cells with a lymphocytic cell pattern. Empirical anti-infective therapy was initiated but later discontinued due to negative bacteriological and viral analysis results. Her condition deteriorated daily, becoming complicated by agitation and epileptic seizures (day 3 since admission), which were treated with levetiracetam. Anti-NMDA receptor antibodies were eventually detected in serum and cerebrospinal fluid (CSF) as part of an extended autoimmune work-up. Oligoclonal bands were not present in the CSF. A 5-day glucocorticoid pulse treatment involving 1 g of methylprednisolone per day was subsequently performed (day 4–8), but without success. Repeated epileptic seizures and confused behaviour dominated the clinical picture. Finally, status epilepticus was detected in the EEG (day 5). Unfortunately, this was refractory to benzodiazepines, lacosamide (400 mg/day), and levetiracetam (2 g/day) (day 6).

### Immune Therapy and Intensive Care

Upon transfer to our intensive care unit, anaesthetic therapy involving ketamine and propofol was initiated (day 9), followed by intravenous immunoglobulin (IVIG) therapy (days 10–12) and plasmapheresis with four plasma exchange sessions (days 13–19). Despite maintaining a burst-suppression pattern for 72 h with ketamine/propofol treatment, status epilepticus could not be interrupted (day 11–13, Fig. 1C). This treatment was followed by isoflurane anaesthesia, which achieved a burst-suppression pattern successfully for 48 h (day 15–16), but could not interrupt the status epilepticus. The dose of levetiracetam was increased to 4 g/day without effect (day 20). Switching from levetiracetam (4 g/day) to brivaracetam (200 mg/day) had no effect (day 23). Organ diagnostics, including tumour screening, revealed a teratoma in the right ovary (Fig. 1B), which was surgically removed (day 30). The patient was treated with rituximab (days 22 and 34). The therapy was escalated to cyclophosphamide due to an unchanged clinical status (day 40). The antiseizure medication was enhanced with successive increases in the dose of perampanel up to 12 mg/day (days 26–38). The EEG recording showed a reduction in ictal activity during perampanel treatment; however, status epilepticus persisted (Fig. 1D).

## Results

### Therapeutic Regimen of Combination Therapy

In light of the literature on the synergistic effects of MCT KD on AMPA receptors in combination with perampanel, we initiated add-on therapy in the form of a fully balanced liquid food containing a mixture of medium-chain triglycerides and docosahexaenoic acid, with a fat-to-carbohydrate-to-protein ratio of 2:1 (Kanso Keto Epi^®^ 2:1), administered via a nasogastric tube (day 41 of hospitalization). According to the manufacturer’s recommendations, the following therapeutic regimen was established: day 1, one bottle; day 2, three bottles; days 3–14, five bottles daily. Each 200 ml bottle of liquid food contains 26 g of MCT, 32 g of fat, 2 g of carbohydrates, 14.2 g of protein, and 350 kcal of energy. Status epilepticus was interrupted on day 7 of MCT treatment. The treatment was continued until day 14 and then discontinued (Fig. 1E). No relapse of status epilepticus was recorded. The patient was successfully extubated and transferred to a rehabilitation clinic. At the time of transfer, she was awake, responsive and able to follow simple instructions and hold simple conversations. The second dose of cyclophosphamide was administered two weeks after transfer to the rehabilitation clinic.

## Discussion

To the best of our knowledge, this is the first successful case of the treatment of super-refractory status epilepticus caused by anti-NMDA receptor encephalitis, in which MCT KD was added to perampanel to improve AMPA modulation. The main component of MCT KD is decanoic acid, which reduces excitatory postsynaptic currents by inhibiting AMPA receptor activity (Chang et al. 2013). The high proportion of decanoic acid in MCTs leads to greater modulation of AMPA receptors, resulting in a more effective antiseizure effect. Therefore, a higher dose of MCT (130 g per day) was required in the presented clinical case. This dose was in line with previously published studies on the use of MCT in drug-resistant epilepsy (Schoeler et al. 2021; Falsaperla et al. 2025).

In our case, we used a combination of immunotherapies. This is why the cumulative effects cannot be clearly ruled out. However, seizure termination was achieved approximately twelve days after rituximab induction treatment and eight days after the first cyclophosphamide cycle, which is too short to establish a causal relationship.

Modulation of the AMPA receptors was effective in this case of anti-NMDA receptor encephalitis, as documented by continuous EEG recordings showing a successive reduction in ictal activity as the perampanel dose increased. One possible explanation for perampanel’s effect in anti-NMDA receptor encephalitis is that it causes cross-linking of antibodies with receptors in the postsynaptic membrane. This results in the internalisation of NMDA receptors and a relative increase in AMPA receptors, leading to increased neural excitation and epileptic seizures (Manto et al. 2010). As an AMPA receptor antagonist, perampanel attenuates nerve hyperexcitability and thus has an antiseizure effect (Akiyama et al. 2019). However, applying the maximal dose of perampanel alone to modulate AMPA was not sufficient to interrupt status epilepticus. Only the additional application of MCT resulted in the termination of super-refractory status epilepticus in this case. Our clinical observation corroborates previous pharmacological studies showing that perampanel and MCT bind to different sites on the AMPA receptor and that decanoic acid can enhance the effectiveness of perampanel (Chang et al. 2013; Augustin et al. 2018).

**Fig. 2:**
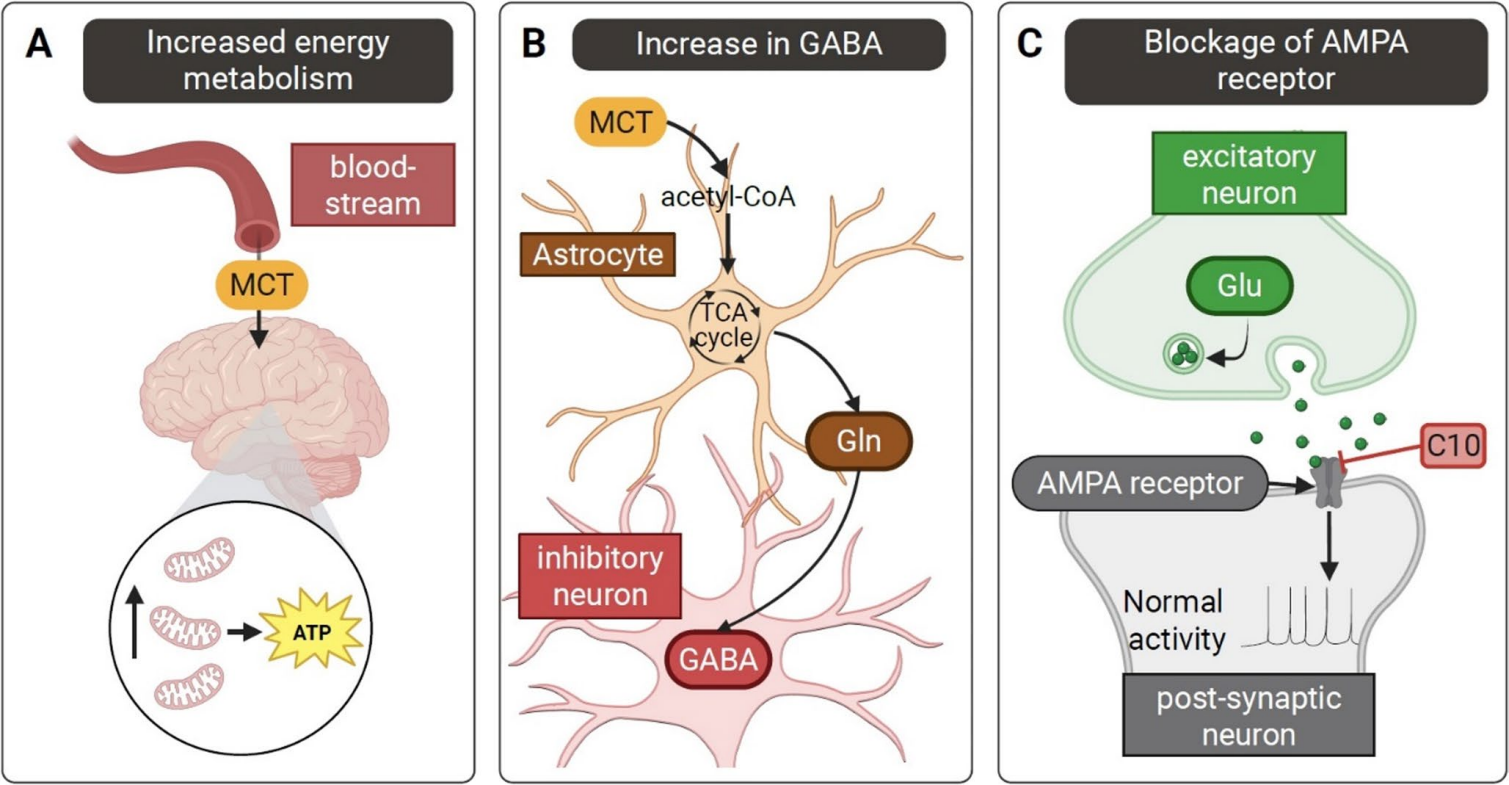
Schematic representation of the mechanisms of action of medium chain triglycerides in epilepsy. (**A**) MCTs readily cross the blood-brain-barrier, resulting in increased mitochondrial biogenesis and improved overall energy metabolism. (**B**) Within the brain, MCTs are preferentially metabolized by astrocytes, enhancing glutamine supply for neuronal GABA synthesis. (**C**) The MCT decanoic acid (C10) acts as a non-competitive voltage- and subunit-dependent antagonist of the AMPA receptor, with a distinct binding site from perampanel

In addition to AMPA modulation, decanoic acid exhibits other pharmacological features that promote antiseizure activity (Fig. 2). It can quickly cross the blood-brain barrier and affect brain homeostasis by improving mitochondrial function, modulating astrocyte activity and increasing the supply of glutamine, thereby increasing neuronal GABA production and inhibiting mTORC1 activity (Shin et al. 2025). Previous studies have shown that MCTs have immunomodulatory properties, such as reducing the production of IL-6 and TNF-α (Yu et al. 2017, 2019). This is relevant to the treatment of anti-NMDA receptor encephalitis because IL-6 and TNF-α are pro-inflammatory biomarkers that are associated with clinical deterioration in this condition (Wang et al. 2019).

## Conclusion

The rational combination of antiseizure therapies and the use of their synergistic effects can improve the outcome of epilepsy treatment in general, and in critical conditions such as super-refractory status epilepticus (Winter et al. 2023). The pathophysiology of seizure-induced brain injury and the origin of status epilepticus must be considered when selecting an optimal treatment strategy (Singh et al. 2022). MCTs may have a synergistic effect when added to perampanel to treat autoimmune status epilepticus. Our clinical data should be validated in larger studies involving patients with autoimmune epilepsy.

## Data Availability

No datasets were generated or analysed during the current study.
